# Energy Bilocalization Effect and the Emergence of Molecular Functions in Proteins

**DOI:** 10.3389/fmolb.2021.736376

**Published:** 2021-12-23

**Authors:** Yann Chalopin, Julien Sparfel

**Affiliations:** Laboratoire EM2C–CNRS and CentraleSupélec, University of Paris-Saclay, Gif-sur-Yvette, France

**Keywords:** protein, rate promoting vibrations, dynamics, vibrations, localization

## Abstract

Proteins are among the most complex molecular structures, which have evolved to develop broad functions, such as energy conversion and transport, information storage and processing, communication, and regulation of chemical reactions. However, the mechanisms by which these dynamical entities coordinate themselves to perform biological tasks remain hotly debated. Here, a physical theory is presented to explain how functional dynamical behavior possibly emerge in complex/macro molecules, thanks to the effect that we term bilocalization of thermal vibrations. More specifically, our approach allows us to understand how structural irregularities lead to a partitioning of the energy of the vibrations into two distinct sets of molecular domains, corresponding to slow and fast motions. This shape-encoded spectral allocation, associated to the genetic sequence, provides a close access to a wide reservoir of dynamical patterns, and eventually allows the emergence of biological functions by natural selection. To illustrate our approach, the SPIKE protein structure of SARS-COV2 is considered.

## Introduction

In his seminal book “What is Life,” Erwin Schrödinger exposed his physical vision of modern molecular biology by priorly emphasizing the intrinsically aperiodic character of living microscopic systems (notably the nucleic acids). His vision arose from the observation that such biological structures failed the physical theories of the time, which were centered, for the most part, on the principle of the periodicity of crystal structures. Indeed, it was the introduction of the concept of regularity (Gross, 1996) (or symmetries) that supported the development of a deep understanding of the laws of quantum mechanics when they are applied to describe matter, its electronic and thermal properties, and its relationship with radiation. Heat in solids corresponds to crystalline vibrations defining quasi-particles called phonons (it is necessary to consider electrons in metals). Phonon transport (heat flux) corresponds to the formation of wave packets that are subjected to dispersion phenomena and propagate according to a mean-free path controlled by various relaxation processes specific to the crystal structure. Heat capacity, thermal expansion or even thermal conductivity are now quantities predicted with substantial precision (Ashcroft and David Mermin, 1976), thanks to the well known physical concept of first Brillouin zone.

Conversely, the transport of energy and/or information in living systems is dominated by intricate cascades of molecular interactions orchestrated by completely irregular structures called proteins. Proteins are macromolecules derived from the translation of RNA into polymer chains of amino-acids (AA), which fold to form unique and irregular structures (Hay and Scrutton, 2012). To operate, proteins need motions. More specifically, this is achieved by the thermal environment: heat constantly maintains proteins dynamics through thermal atomic displacement fluctuations from the thermal bath (in most cases, the solvent), at timescales ranging from the fs (e.g., proton transfer) to the ms timescales (e.g., conformational changes) (Damry et al., 2019). This dynamics plays for instance a determining role in enzyme catalysis (Wolf-Watz et al., 2004; Yang and Bahar, 2005; Henzler-Wildman et al., 2007; Boekelheide et al., 2011; Callender and Dyer, 2015; Keul et al., 2018), allostery (del Sol et al., 2007), and molecular recognition. What we want to stress here is that a protein is an object that results from a regular system’s distortion towards a disordered/inhomogeneous structure or topology, because we believe that the coexistence between structural order and disorder produces dynamical patterns that have been perceived as functional throughout natural selection (Keul et al., 2018; Borgia et al., 2018). Why could it be so? Despite the demonstrated importance of protein dynamics, the interplay between the genetic sequence and the time sequence of functional motions remains widely undiscovered (Pancsa et al., 2016). Current efforts in contemporary molecular biology focus particularly on finding ways to describe proteins as structure-driven dynamical entities. While it has long been a question of how the structure dictates the function, the current paradigm should be shifted towards quantitatively describing function more as a result of dynamical properties, which are themselves driven by shapes (Ishima and Torchia, 2000). Unlike solids, we claim here from the basis of mathematical arguments that living organisms ultimately rest on the ability of evolution to shape irregular molecular structures (Keul et al., 2018) to produce functional dynamical patterns. Unfortunately, developments in molecular biology and condensed matter physics have made their way apart. Consequently, few studies have apprehended proteins’ dynamics with a starting point the entanglement between disorder (shape) and propagation phenomena sustained by the environment (heat). The transport of non-interacting electrons in disordered structure has been extensively investigated (Filoche and Mayboroda, 2012; Arnold et al., 2016; Lemut et al., 2020) [the main known effect of which is Anderson localization (Anderson, 1958)] while very few works have formalized localization phenomena in phonon systems (Keppens et al., 1998; Seyf et al., 2017; Juntunen et al., 2019; Zhou et al., 2020). In this letter, we are inspired by a powerful mathematical tool (Filoche and Mayboroda, 2012) to describe a theory which offers the possibility to understand the entanglement between microscopic lattice vibrations and propagation phenomena in a complex topology, in order to give meaning to the dynamics of proteins and enzymes. The following study is based on a central assumption, which is that such dynamical systems, potentially functional, arise from sets of motions involving the largest number of different domains, themselves associated to the most distinct characteristic times and amplitudes to be selected (McDonald et al., 2013). This leads us to address proteins’ functioning by formulating a question in physical terms: how to reconstruct the functional dynamics -that is which linear combination of normal modes-of a macro-molecule by associating each characteristic time to a spatial domain, from the knowledge of the 3D structure solely ? Finally, once this quantity is mapped on proteins’ topology, what is revealed ?

## Materials and Methods

### A General Description of the Protein Dynamics

**FIGURE 1:**
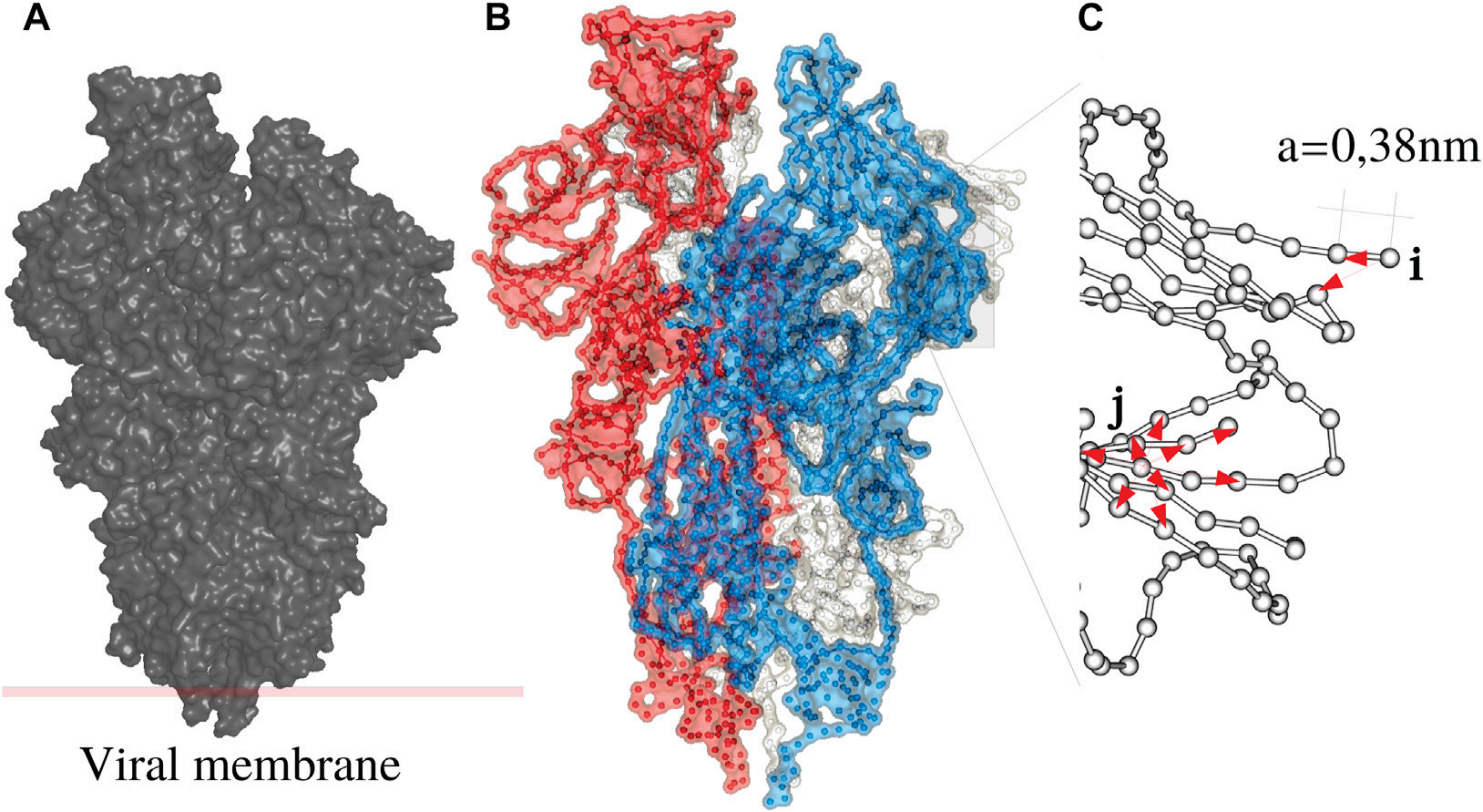
The Spike (S) protein as an example of a disordered system **(A)** consists of three monomers **(B)**, white, blue and red. Each monomer is a periodic chain of amino acids with a period of 0.38 nm **(C)**. A protein is a folded chain where order and disorder intermingle.

Any periodic sequence composed of invariantly coupled degrees of freedom universally leads to dispersive wave transport. Like any other folded polymer system, a protein can be first approximated as a linear chain of AA bonded between the *C*_*α*_ atoms (backbone) as shown on Figure 1. However, these covalent interactions are completed by noncovalent bonds between the side chains: proteins fold, and consequently, each degree of freedom of the BB has cohesive energy that is no longer spatially homogeneous Figure 1. Based on these physical considerations, we express the lagrangian of the system as,

$$L=\overset{N}{\underset{i=1}{\sum }}\frac{1}{2}{\dot{X}}_{i}^{2}-\underset{1\leq i<j\leq N}{\sum }\frac{1}{2}\alpha_{ij}{\left(X_{i}-X_{j}\right)}^{2}$$ (1)

which allows to extract the harmonic equations of motion of such a dynamical system as

$$\omega^{2}X_{i}=\left(\overset{N}{\underset{j=1}{\sum }}\alpha_{ij}\right)X_{i}-\overset{N}{\underset{j=1}{\sum }}\alpha_{ij}X_{j}$$ (2)

where *ω* stands for the harmonic pulsation, *X*_*i*_ is the displacement of atom *i* from its equilibrium position *x*_*i*_. The *α*_*ij*_ are constants characterizing the strength of the bond between atoms *i* and *j*. We see from this eq. that disorder (irregularity) is reflected by the quantities *α*_*ij*_ which account for the nature of the interactions (ex. angular or distant dependent) as well as the number of “neighbors” included in the sum. We introduce the matrix (operator):

$$-\Delta :\left(\delta_{ij}\right)=\left\{\begin{array}{cc} \overset{N}{\underset{k=1}{\sum }}\alpha_{ij},\; & \text{if}\;i=j.\\ -\alpha_{ij},\; & \text{otherwise}. \end{array}\right.$$ (3)

such that the equations of motion are casted into an eigenproblem in the form of a Laplace equation:

$$-\Delta X=\omega^{2}X$$ (4)

We consider vibrations defined at the zone-edge, by the wavenumber *k* = *π*/*a*: ${\tilde{X}}_{j}=e^{i\frac{\pi }{a}x_{j}}X_{j}={\left(-1\right)}^{j}X_{j}$ . We introduce a real constant *C*, taken slightly greater than the greatest eigenvalue $\omega_{\max}^{2}$ of − Δ and write

$$\left(C-\omega^{2}\right){\tilde{X}}_{i}=\left(C-\overset{N}{\underset{j=1}{\sum }}\alpha_{ij}\right){\tilde{X}}_{i}+\overset{N}{\underset{j=1}{\sum }}\alpha_{ij}{\left(-1\right)}^{j-i}{\tilde{X}}_{j}$$ (5)

with an operator *L*_*h*_ defined by:

$$L_{h}\tilde{X}=\left(C-\omega^{2}\right)\tilde{X}$$ (6)

### Localized Fast and Slow Motions and Bilocalization Effects

A first frequency localization landscape (LL) *u*_*h*_ is obtained from

$$L_{h}u_{h}=\left(1\right),$$ (7)

where (1) = (1,‥,1)^*T*^, which is termed high frequency LL for its ability to capture the localization of the highest frequency modes (Chalopin et al., 2019). In the case of a continuous elliptic differential operator *H* on a domain Ω, it has been shown that the localization landscape *u* defined as *Hu* = 1 on Ω satisfies $\frac{\left|\Psi \left(x\right)\right|}{\|\Psi {\|}_{\infty }}\leq Eu\left(x\right)$ where Ψ is an eigenfunction of *H* with eigenvalue *E*. We would then expect in our case to have $\frac{{\tilde{X}}_{i}}{\|\tilde{X}{\|}_{\infty }}\leq \left(C-\omega^{2}\right)u_{i}$ for every 1 ≤ *i* ≤ *N*. Unlike in the continuous case this is not generally true. The maximum principle is indeed not true for the *L*_*h*_ operator. Components of $L_{h}^{-1}$ are then not strictly positive and the inequality is false in general. However we numerically find that this inequality is fulfilled on most cases. Therefore, high-frequency modes are expected to be strongly localized on the maxima of *u*_*h*_ or equivalently on the minima of *W*_*h*_ = 1/*u*_*h*_.

In a discrete disordered system the dispersion relation is no longer monotonous: wave-vectors at the zone edge are no longer systematically associated with the highest frequencies. For this reason low frequency modes are expected to be localized too. We introduce a complementary localization landscape that captures low frequency localization at the zone edge.

We reconsider the dynamics of the system written as:

$$\left(\omega^{2}+\epsilon \right){\tilde{X}}_{i}=\left(\epsilon +\overset{N}{\underset{j=1}{\sum }}\alpha_{ij}\right){\tilde{X}}_{i}-\overset{N}{\underset{j=1}{\sum }}\alpha_{ij}{\left(-1\right)}^{j-i}{\tilde{X}}_{j}$$ (8)

and introduce the operator *L*_*l*_:

$$L_{l}\tilde{X}=\left(\omega^{2}+\epsilon \right)\tilde{X}$$ (9)

which is positive definite with *ϵ* a small constant. The low frequency localization landscape *u*_*l*_ is straightforwardly obtained from:

$$L_{l}u_{l}=\left(1\right)$$ (10)

**FIGURE 2:**
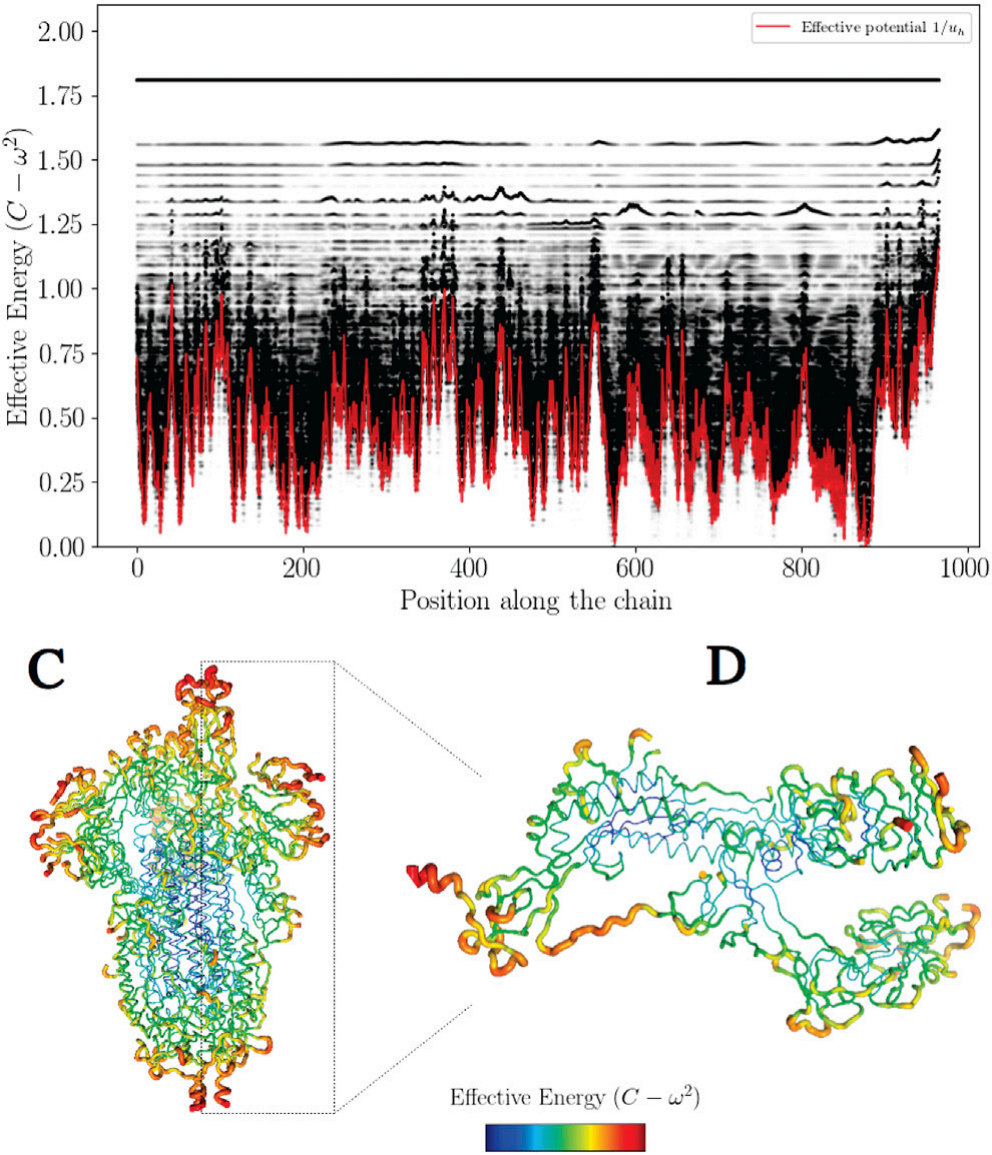
The effective confining potential of high frequencies. The high frequency confinement predicted by *W*_*h*_ is in agreement with the structure of the density of vibrational states (here $X_{i}^{2}$ ) on a band corresponding to the highest frequencies **(A)**. 3D representation of *W*_*h*_ for the complete protein (S) **(B)** with a zoom on the first monomer **(C)**.

**FIGURE 3:**
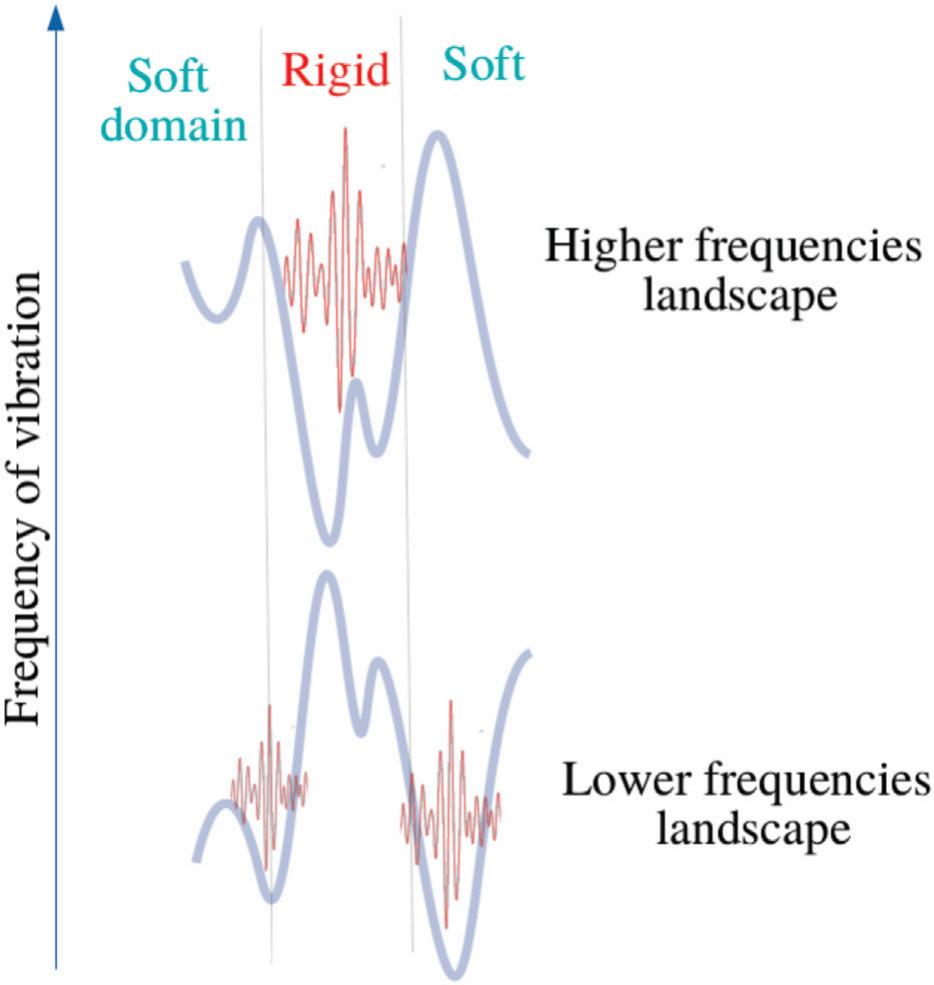
Effective confinement potentials.

As previously the inequality $\frac{{\tilde{X}}_{i}}{\|\tilde{X}{\|}_{\infty }}\leq \left(\omega^{2}+\epsilon \right)u_{i}$ is not generally true but happens to be true on most cases. On Figure 2 the effective confining potential *W*_*l*_ = 1/*u*_*l*_ (red) of the Spike protein of SARS-Cov2 is displayed. One can clearly see that low frequency modes are localized on the minima of *W*_*l*_. As it can be seen on Figure 3, the 2 localization landscapes happen to be complementary: the minima of the high frequency LL are located on the same sites as the maxima of the low frequency LL and conversely. This can be explained by a simple relation between operators *L*_*h*_ and *L*_*l*_:

$$L_{h}=\left(C+\epsilon \right)I_{N}-L_{l}$$ (11)

where $I_{N}$ denotes the *N* × *N* identity matrix. *L*_*l*_ being symmetric and positive definite, there exists an orthogonal matrix *O* and a diagonal matrix Σ with strictly positive eigenvalues such that:

$$L_{l}=O\Sigma O^{T}$$ (12)

Note that matrix *O* can be chosen such that it is the matrix of change of basis from the canonical basis to the basis of envelopes at *k* = *π*/*a* (which are eigenvectors of *L*_*l*_). Localization landscapes are defined as:

$$\left\{\begin{array}{c} L_{h}u_{h}=\left(1\right)\;\\ L_{l}u_{l}=\left(1\right)\; \end{array}\right.$$ (13)

We now use the diagonalization of *L*_*l*_ in these two expressions:

$$\left\{\begin{array}{c} \left(\left(C+\epsilon \right)I_{N}-O\Sigma O^{T}\right)u_{h}=\left(1\right)\;\\ O\Sigma O^{T}u_{l}=\left(1\right)\; \end{array}\right.$$ (14)

$$\;\Rightarrow \;\left\{\begin{array}{c} \left(O^{T}\left(C+\epsilon \right)I_{N}-\Sigma O^{T}\right)u_{h}=O^{T}\left(1\right)\;\\ \Sigma O^{T}u_{l}=O^{T}\left(1\right)\; \end{array}\right.$$ (15)

$$\;\Rightarrow \;\left\{\begin{array}{c} \left(\left(C+\epsilon \right)I_{N}-\Sigma \right)u_{h}^{*}={\left(1\right)}^{*}\;\\ \Sigma u_{l}^{*}={\left(1\right)}^{*}\; \end{array}\right.$$ (16)

where we have denoted v* = *O* ^*T*^ *v* for any column vector *v* and we have used the fact that *O* ^*T*^ and $\left(C+\epsilon \right)I_{N}$ commute. Thus we have:

$$\left(\left(C+\epsilon \right)I_{N}-\Sigma \right)u_{h}^{*}=\Sigma u_{l}^{*}$$ (17)

Matrices Σ and $\left(C+\epsilon \right)I_{N}-\Sigma$ being diagonals we have for every 1 ≤ *i* ≤ *N*:

$${\left(u_{h}^{*}\right)}_{i}=\frac{\Sigma_{ii}}{C+\epsilon -\Sigma_{ii}}{\left(u_{l}^{*}\right)}_{i}$$

where we have denoted (*v*)_*i*_ the *i*th component of a column vector *v*. The Σ_*ii*_ are eigenvalues of matrix *L*_*l*_ so we have $\Sigma_{ii}=\omega_{i}^{2}+\epsilon$ for every 1 ≤ *i* ≤ *N*. Eventually we obtain a very simple relation between components of the localization landscapes in the basis of envelopes at *k* = *π*/*a*:

$${\left(u_{h}^{*}\right)}_{i}=\frac{\omega_{i}^{2}+\epsilon }{C-\omega_{i}^{2}}{\left(u_{l}^{*}\right)}_{i}$$ (18)

Recall that we choose constant *C* slightly greater than the greatest eigenvalue $\omega_{\max}^{2}$ of − Δ. Therefore, for low frequency *ω*_*i*_ Eq. 18 implies ${\left(u_{h}^{*}\right)}_{i}\ll {\left(u_{l}^{*}\right)}_{i}$ . For high frequency *ω*_*i*_ Eq. 18 implies ${\left(u_{l}^{*}\right)}_{i}\ll {\left(u_{h}^{*}\right)}_{i}$ .

### How to Look at the Structural Disorder as Quantum-Like Confinement Potentials

Here we describe how the inverse quantities for both landscapes (1/*u*) shall be regarded as effective confining potentials (CP). This important quantity (CP) has been priorly introduced to succesfully describe confinement of electronic wavefunctions (Arnold et al., 2016).

We demonstrate that in discrete phonon systems, any localization landscape aforementioned (e.g *u*_*l*_) also leads to the apparition of an effective potential (*W*_*l*_ = 1/*u*_*l*_) in the equations of motion (Eq. 25), which take a form similar to the Schrödinger equation describing a free particle (e.g electron) in an irregular quantum well.

We start with the equation defining the operator *L*_*l*_:

$$L_{l}\tilde{X}=\left(\omega^{2}+\epsilon \right)\tilde{X},$$ (19)

which can be expanded in the continuous limit as

$$-\left[div\left(A\nabla \right)+V\right]\tilde{X}=\left(\omega^{2}+\epsilon \right)\tilde{X}$$ (20)

We introduce $\tilde{X}=u\phi$ to account for the modulation of the mode with an envelop defined by *u* = *u*_*l*_. This provides:

$$-div\left(Au\nabla \phi \right)-div\left(A\phi \nabla u\right)+Vu\phi =\left(\omega^{2}+\epsilon \right)u\phi .$$ (21)

Using the Leibniz Formula

$$div\left(a\vec{x}\right)=a\nabla \cdot \vec{x}+\vec{x}\cdot \nabla a$$ (22)

we obtain

$$\begin{array}{c} -u\nabla \cdot \left(A\nabla \phi \right)-\left(A\nabla \phi \right)\cdot \nabla u-\phi \nabla \cdot \left(A\nabla u\right)-A\nabla u\cdot \nabla \phi +Vu\phi =\left(\omega^{2}+\epsilon \right)u\phi \\ -u\nabla \cdot \left(A\nabla \phi \right)-2A\nabla u\cdot \nabla \phi +\phi \left(-\nabla \cdot \left(A\nabla u\right)+Vu\right)=\left(\omega^{2}+\epsilon \right)u\phi \\ -\nabla \cdot \left(A\nabla \phi \right)-2A\frac{\nabla u}{u}\cdot \nabla \phi -\frac{\phi }{u}=\left(\omega^{2}+\epsilon \right)\phi \end{array}$$ (23)

where we used the fact that − ∇⋅ (*A*∇*u*) + *Vu* = 1. Eventually we get

$$\left[-\frac{\nabla \cdot \left(u^{2}A\nabla \right)}{u^{2}}+\frac{1}{u}\right]\phi =\left(\omega^{2}+\epsilon \right)\phi$$ (24)

replacing by the vector *X*, we obtain

$$-\frac{1}{u_{l}^{2}}\;\text{div}\left(u_{l}^{2}A\nabla X\right)+\frac{X}{u_{l}}=\omega_{n}^{2}X.$$ (25)

To illustrate this feature, the high frequency confinement potential has been color-coded in 3D on Figure 2.

**FIGURE 4:**
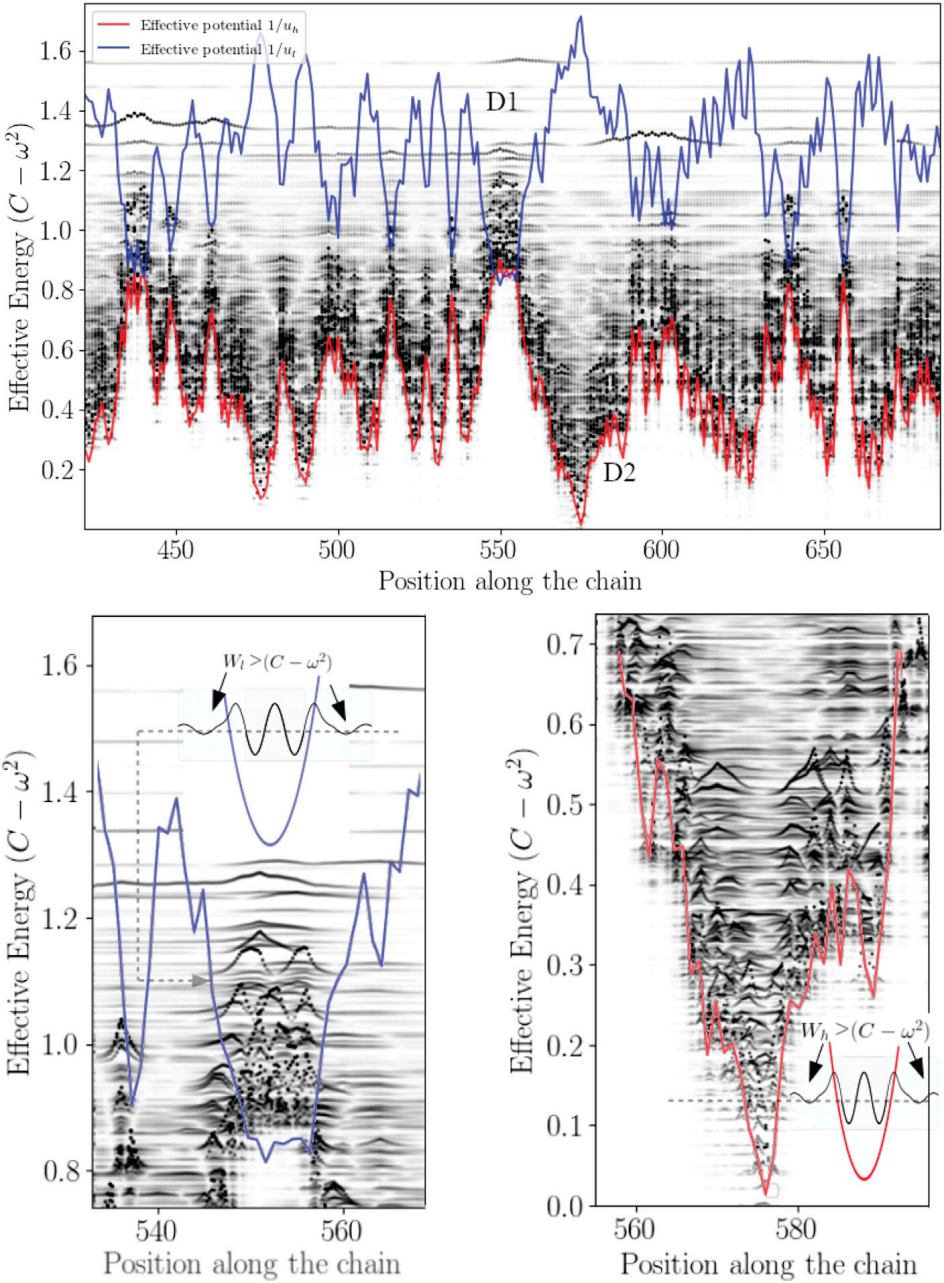
Bilocalization effect The modulus of the normal modes are plotted and shifted according to their eigenvalues by *C* − *ω* ^2^ (dark points). The confinement potentials corresponding to high (red) and low frequency modes (blue) are spatially complementary. The modal amplitude decays exponentially inside the potential.

This analogy leads to introduce the concept of effective barrier through the quantity *W*_*h*_ − (*C* − *ω* ^2^). Consequently, phonons wavenumbers become purely imaginary where (*C* − *ω* ^2^) − *W*_*h*_ < 0, that is wave amplitudes vanish with exponential decays (Figure 3 and Figure 4). This later aspect is crucial for understanding how a protein operates from the knowledge of its energy partition.

In this work, most the discussion is exemplified on the SPIKE (S) protein of SARS-CoV-2, which is a molecule that plays a key role in the virus replication (Turoňová et al., 2020). Without providing too much details, one can simply recall that it serves as a receptor to bind to the angiotensin 2 converting enzyme of the host receptors, while initiating a binding with the cell membrane before fusing with the latter (S) is formed by three chains (a trimer) of polypeptide polymers (Figure 1B). Each chain (monomer) consists of a sequence of amino acids regularly spaced by 0.38 nm (see Figure 1) while the folded structure (3D) form a very irregular object. Its dynamical properties (vibrations) play a crucial role in understanding how the virus interacts with its host (Di Paola et al., 2020). The normal modes are calculated from − Δ considering a parameter free elastic network model (pfENM-GNM) (Yang et al., 2009) as shown in Figure 3. The effective high-frequency localization potential obtained from Eq. 7 is also embedded in the figure to highlight confinement effects by the potential: just like Anderson localization, phonon confinement manifests itself by an exponential modal decay upon the effect of an effective barrier. However, *W*_*h*_ does not completely describe the whole spectrum (*i.e*., low-frequency motions). When the effective energy *C* − *ω* ^2^ rises above the maxima of the potential *W*_*h*_, novel localizing regions appear where the dynamics were priorly forbidden. Interestingly, each site that is “frozen” within a given frequency band switches to a dynamically active domain, once driven in the complementary frequency band. Figure 3 and Figure 4 illustrate this complementary relationship between *W*_*l*_ and *W*_*h*_ as predicted by Eq. 11. These potentials are said reciprocal, and the dynamical result of their interplay is termed bilocalization.

This observation leads to the next question: estimating the density of states (DOS) without solving the eigenvalue problem. Weyl’s law offers an asymptotic estimate of the DOS, and it has been successfully employed in the case of electronic wavefunctions (Arnold et al., 2016) where the number of modes with energy lower than *E* is estimated as

$$\begin{array}{cc} N\left(E\right) & \approx N_{W}\left(E\right)\\ & ={\left(2\pi \right)}^{-n}\int_{k^{2}+W\left(x\right)\leq E}{\mathrm{d}}^{n}x{\mathrm{d}}^{n}k \end{array}$$ (26)

Where *W* corresponds to the localization landscape for electrons in a random potential and *E* the effective energy. In our case, since normal modes are localized in space, by Fourier properties their width in *k*-space is expected to be large. Thus, one may assume that all wave vectors are accessible to a state localized on a scale of few DOF. Instead of summing over the volume of phase space such that $k^{2}+W_{h}\left(x\right)\leq C-\omega_{0}^{2}$ we sum over all wave vectors of the Brillouin zone and only impose a condition on position: $W_{h}\left(x\right)\leq C-\omega_{0}^{2}$ . To go from a continuous integral to a discrete sum we use $\mathrm{d}k\to \frac{\pi }{Na}$ , ⅆ*x* → *a* and *∫* → *∑*. The estimation is then:

$$\begin{array}{cc} N\left(C-\omega_{0}^{2}\right) & \approx N_{W_{h}}\left(C-\omega_{0}^{2}\right)\\ & =\frac{1}{2\pi }\underset{\underset{W_{h}\left(x_{i}\right)\leq C-\omega_{0}^{2}}{x_{i},k_{n}}}{\sum }\frac{\pi }{Na}a\\ & =\frac{1}{2N}\underset{\underset{W_{h}\left(x_{i}\right)\leq C-\omega_{0}^{2}}{x_{i}}}{\sum }\underset{k_{n}}{\sum }1\\ & =\frac{1}{2N}\underset{\underset{W_{h}\left(x_{i}\right)\leq C-\omega_{0}^{2}}{x_{i}}}{\sum }2N\\ & =\underset{\underset{W_{h}\left(x_{i}\right)\leq C-\omega_{0}^{2}}{x_{i}}}{\sum }1 \end{array}$$ (27)

**FIGURE 5:**
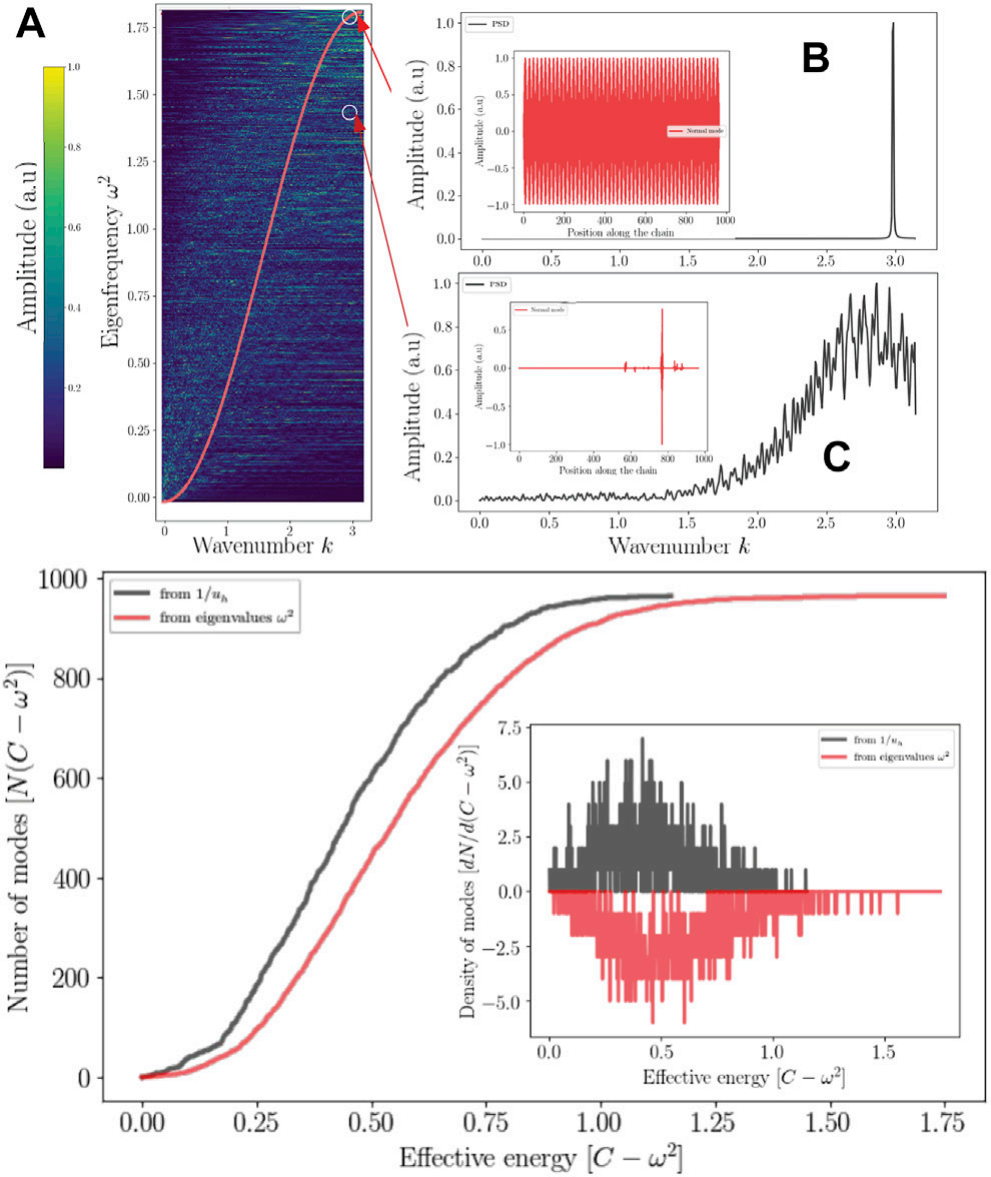
Localization and reciprocal space Top: Localized wave and the reciprocal spaces **(A)** Dispersion relation of (S) compared to a perfect chain of the same size (red) **(A)**. The range of the power spectral density of a localized mode is enlarged **(C)** compared **(B)** to a delocalized mode (standing wave). Bottom: The eigenvalues counting function predicted by the effective potential (dark) as well as that obtaiFed from the eigenvalues of Λ. Their derivative correspond to the DOS (Inset).

*N*(*x*) corresponds to the number of modes having a frequency such that *C* − *ω* ^2^ ≤ *x* and should be termed the eigenvalue counting function (Arnold et al., 2016). Figure 5 shows comparison of this estimation and the true counting function $N\left(C-\omega_{0}^{2}\right)$ for (S).

Eq. 27 tends to overestimate the number of high-frequency modes but gives a fair approximation of the density of states. What emerges from these results, which can be generalized for any discrete disordered dynamical system, is that.

1–Disordering a molecular crystal produces two localized energy bands predicted by a double wave confinement potential. These potentials have the particularity of being complementary in the sense that (Eq. 1) temporally, they predict the locations of slow (*u*_*l*_) and fast (*u*_*h*_) motions, moreover (2) the domains associated with each temporally distinct landscapes are spatially complementary: their association reproduces the molecular domain as a whole (Figure 4).

2–The localization potential of slow motions is a reading of the local rigidity (see Sup. Mat.). Thus, any mode of effective energy higher than the quantity *∑α*_*ij*_ is no longer influenced by *W*_*l*_. Above a threshold corresponding to the maximum of the *W*_*l*_, the localization potential *W*_*h*_ takes over.

3–The localized modes are subject to uncertainty Δ*x* ⋅Δ*k* ≥ 2*π*, so any strong localization of the energy of a mode implies a broadening of the spectrum associated with the spatial frequencies. Disorder effect on dispersion can be approximated by the modes’ projection at the edge of the BZ. Figure 5 illustrates this aspect.

4–Complementarity implies that whatever the characteristic time considered, for each DOF, there is always a cutoff frequency below which the dynamics will be inhibited and vice-versa. The concept of bi-localization implies that there are no frozen domains. Being active or at rest, this only occurs on a limited frequency band. This description is consistent with the equipartition theorem of energy.

## Discussion

**FIGURE 6:**
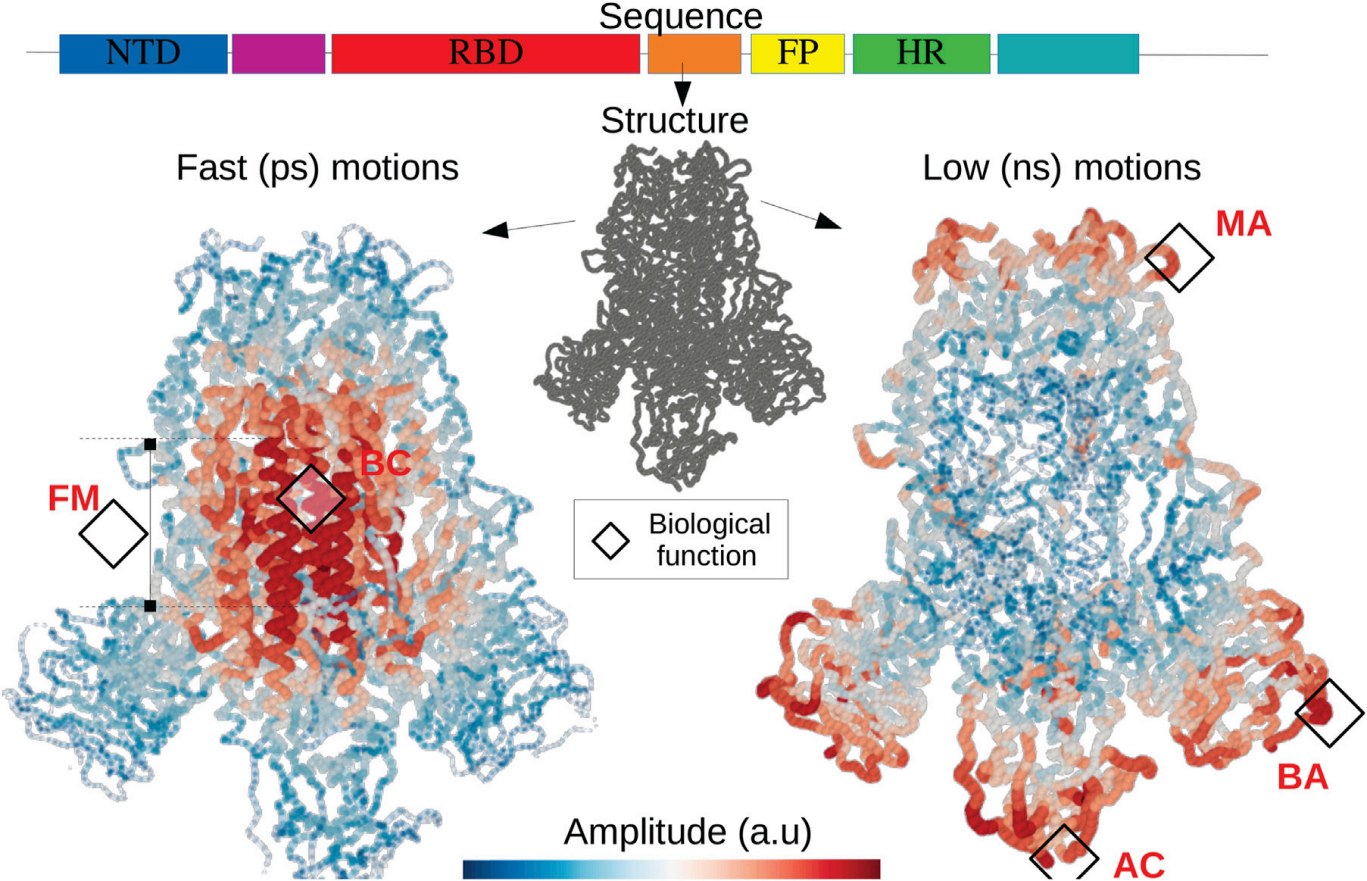
The fold encoded functional dynamics of SARS-Cov2 Spike protein The translation of the RNA into a protein structure gives rise to two hidden landscapes of localized modes corresponding to fast and short timescales. The localization landscapes allow to predict functional domains such as the fusion machinery (FM) as well as the bond cleavage (BC) (Walls et al., 2020), the scanning (Turoňová et al., 2020) as well as the binding (AC) with the host cell surface (Huang et al., 2020), the binding with potential neutralizing antibodies (BA). Other soft domains, allow to facilitate motions interfering with antibody (MA) (Turoňová et al., 2020) and subjected to several mutations. This illustrates the quantitative relationship between the expression of the DNA code and the physical origin of functional domains.

Whether a many-body disordered system can be thermalized is a question that has remained unanswered for a long time (Wang et al., 2020). We show here that any coherent transport regime, integrated into a discrete disordered structure, produces two distinct broadband localization effects similar to Anderson’s localization but contrary to the case of non-interacting electrons (Lyra et al., 2015), strong localization occurs at the zone edge for high and low energies. Such partition of energy spectrum into two spatially complementary subdomains avoids the freezing of a DOF when the system is set in contact with a thermostat. Therefore bilocalization establishes a prerequisite for energy equipartition in phonon systems. In other words, discrete disordered systems at the equipartition produce a rich dynamics, *i.e.*, involving motions in many subdomains and this at very different characteristic times. These two localization effects imply a broadening of the spatial frequency spectrum resulting in zero group-velocity modes (Arregui et al., 2019), which dramatically reduces the heat flux. Although this subject is out of the scope of the paper, the reduction in thermal conductivity observed in disordered solids (Keppens et al., 1998; Seyf et al., 2017; Juntunen et al., 2019; Zhou et al., 2020) must therefore be understood as the consequence of a projection of the phonon dispersion relation at the zone-edge. Nature, for its part, can take advantage of such dynamical effects associated to wave localization. Proteins appear to be thermally driven nanomachines: slow modes at the *ns* to *ms* timescales (also termed conformational dynamics) correspond to conformation changes of large molecular domains, they are of fundamental importance and therefore extensively studied (Bahar et al., 1999; Henzler-Wildman et al., 2007; Callender and Dyer, 2015; Damry et al., 2019). Their biological functions often lie in the establishment of characteristic times associated with the regulation of chemical reactions such as enzymatic catalysis (Wolf-Watz et al., 2004) as well as allostery (del Sol et al., 2007). Large scale dynamics is well predicted by the landscape *W*_*l*_. The application of this framework of analysis on (S) allows highlighting the flexibility at the level of contact with the viral membrane, which allows the latter to orient itself effectively to scan the host cell surface as well as to bind ACE2 (Turoňová et al., 2020). The conformational movements called “up” and “down” are also well described (Huang et al., 2020; Walls et al., 2020) by the landscape *W*_*l*_. The faster functional motions (on the ps time range) are termed rate-promoting vibrations (RPV) (Henzler-Wildman et al., 2007; Hay et al., 2010; Hay and Scrutton, 2012; Schramm and Schwartz, 2018). They play the role of increasing the kinetics of chemical reactions at the level of active sites. Their physical origin has been extensively debated (Schramm and Schwartz, 2018; Antoniou and Schwartz, 2001). The geography of hot-spots hosting fast localized motions is very well reproduced by the *W*_*h*_ potential (Chalopin et al., 2019). For (S), these hot spots reveal cavities corresponding to the fusion machinery. The bond cleavage occurring at the fusion-peptide region (Walls et al., 2020) collocates with a domain of fast compressive vibrations (as seen on Figure 6 in red) to activate the protein for membrane fusion. All localization hot-spots revealed by both landscapes correspond to functional domains considered as potential therapeutic targets (Di Paola et al., 2020; Huang et al., 2020; Walls et al., 2020). These two-folded localized dynamics, evolving at two distinct timescales, are likely functionally relevant, and at least universally present in enzymes and all proteins in general. For a function to emerge, something has to be selected. If all the DOFs of a molecule, such as a non-folded polymer, move coherently in time and space, then there is no machinery to select. It is the association of as many frequencies as possible with different domains that produces the richest dynamics. Bilocalization can therefore be perceived as a way to increase the variability of the distribution of these characteristic times over different domains of the molecule.

This demonstrates that the coexistence of order and disorder tends to produce systems capable of evolving with an enhanced variability of dynamical patterns, offering to evolution a possibility to achieve molecular functionalities by selecting the 3D topology. Is that all ?

This particular description of the energetic properties exposed here must be further discussed and confronted with previous studies. It is therefore appropriate to present in particular the work of Leitner et al. (2015) with whom we share this idea that the origin of the functional properties of proteins and enzymes is intrinsically associated with the way in which a given structure is able to diffuse heat in the molecular scaffold (Leitner and Yamato, 2020). His approach consists in calculating the local (i.e., residue scale) diffusion on the basis of atomistic simulations such as molecular dynamics. The considerable value that Leitner brings to this problem lies in the ability to relate the transport coefficients (Yu and Leitner, 2005) to the reaction rates by the so-called Master equation (Leitner et al., 2015). We quote here in a non-exhaustive way some works that resonate perfectly with our description. First of all, we fully agree that in these systems, the diffusion of energy does not take place homogeneously, but rather along distinct paths connecting sub-regions of the protein (what we call here the valleys of confinement potential) giving rise to a transport by percolation (Leitner and Yamato, 2020; Leitner, 2008). That is, energy can propagate from residue A to residue B if and only if these residues are part of a connex network defined by their mechanical coupling (cohesive energy). We believe that hoping transport occurs between different localization valleys through a universal phenomenon knbown as tunneling effect by evanescent waves. Secondly, we share the conclusion that the transport is anisotropic (Leitner, 2008) since these localization spots are not uniformly distributed in the protein.

Furthermore, Leitner’s works also showed that energy percolation between a catalytic residue and other distant residues allows the modulation of chemical bonds through thermal fluctuations (which results in the fluctuations in the length of hydrogen bonds) and that the corresponding network of connected residues correlate well with the mapping of energy flow (Leitner and Yamato, 2020).

We also agree that the role of contacts is preponderant (Leitner et al., 2019; Yamashita et al., 2018). As shown in our model, re-strengthening the cohesive energy locally induces the formation of hot spots (Chalopin, 2020) which can be used as a “hub” (see for instance the litterature dealing with graph centrality).

**FIGURE 7:**
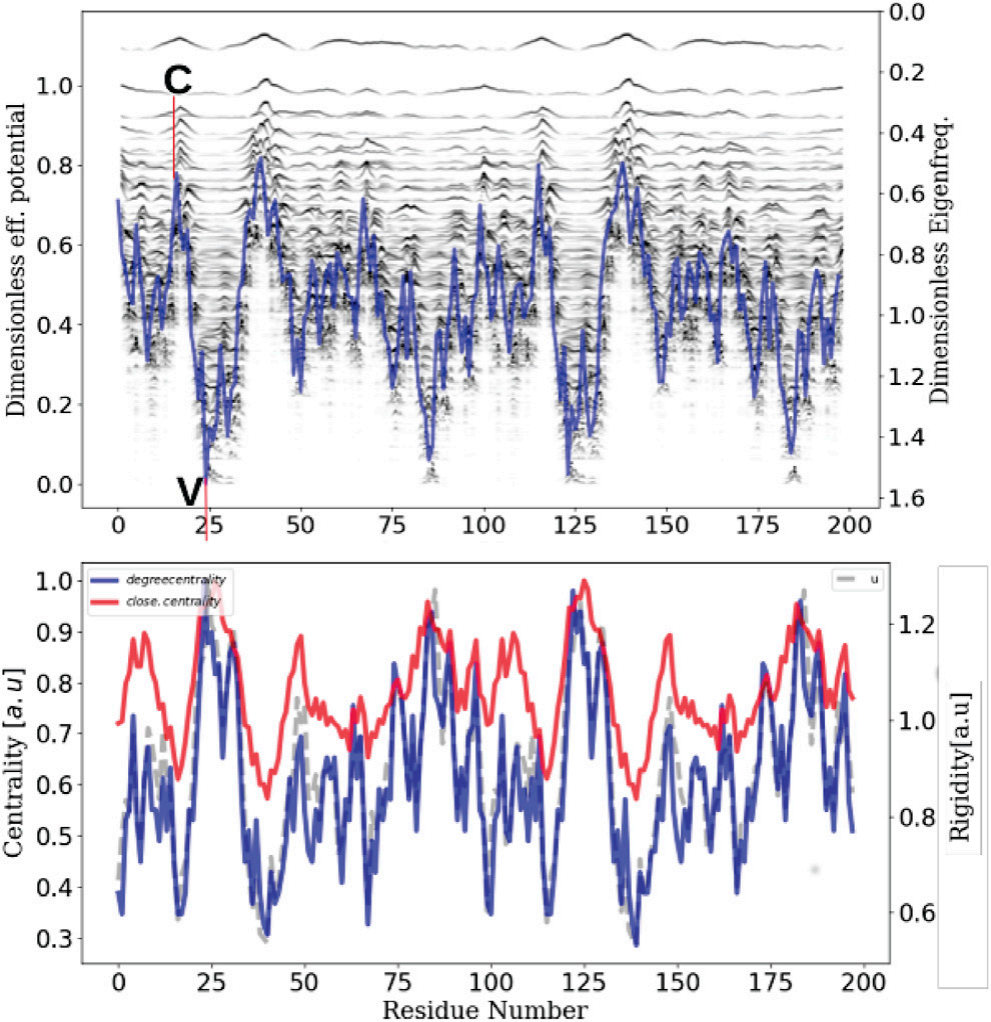
Network centrality, rigidity and localization landscape The inherent ability of a protein structure to generate confining potentials (top plot)–which in turn will define energy transport properties–is intrinsically linked to its rigidity (grey), which in turn can be seen as a modulation of connectivity or centrality (red and blue).

To demonstrate the adequacy between these well established results and our fundamental description of these transport phenomena, we have performed non equilibrium molecular dynamics simulations using the velocity verlet technics coupled to a pfENM hessian (ANM) (Yang et al., 2009). To simplify our discussion as much as possible without diminishing the general scope of our remarks, we have chosen a much smaller system than SPIKE trimer such as the HIV1-protease enzyme (PDB id:1rx7) and calculate the confinement potential of the dimer. The corresponding plot (top of Figure 7) allows us to identify the valleys of the confinement potential in which the energy must remain confined or a contrario the collars which will allow the isolation of the high frequency domains. We have chosen two sites (a collar C) and a valley (V, corresponding to the active sites) and we parametrically excite an AA in each of these domains, at the fastest frequency of the system (here 3 *THz*).

**FIGURE 8:**
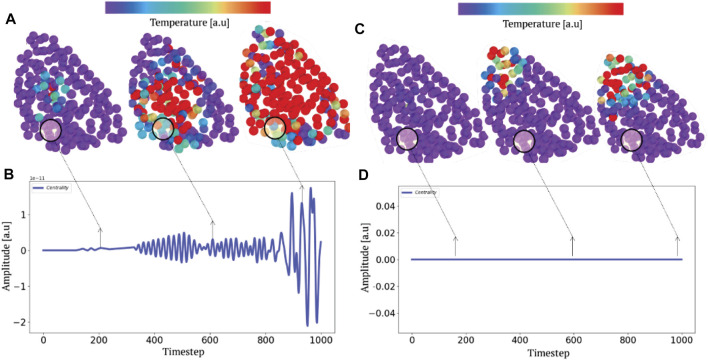
Selective parametric excitation and anisotropic heat transport. A parametric excitation at the highest frequency **(A)** and **(C)** produces a non-equilibrium transport phenomenon. Tracking the progression **(B,D)** of heat fields by molecular dynamics depending on whether the system is excited in a region of high **(A)** or low **(B)** potential (or connectivity) localization illustrates the anisotropic nature of the thermal relaxation.

From this selective excitation on the AAs, we follow temporally how the locally injected energy propagates in the scaffold of the protein (Figure 8). On the first plot (right), we notice that when we excite the system in a domain that does not correspond to a confinement potential well, the energy does not propagate in the system but remains localized at the level of the excitation (C and D). However, when this excitation is performed at the level of the active sites (left), which correspond to the most confining domains, we notice that the energy propagates in an dramatically more efficient way in the whole system (A and B). This numerical experiment allows to emphasize unequivocally that the transport is indeed anisotropic and that this anisotropy is prescribed by the localization landscape, thus generalizing Leitner’s results under a broader physical picture. In order to compare our approach to the state of the art, we calculated the degree of centrality of the system and compared it to the localization landscape (bottom of Figure 7), thus revealing a correlation factor between the two quantities that amounts to 0.9. This last result allows us to claim that rigidity, connectivity or degree of centrality are quantities that relate to the same fundamental physical effect, that is the vibrational confinement potential. Thus, it becomes possible to associate these quantities to strongly anisotropic energy transport properties, and that this is this particular transport mechanism (associated to the percolation through confined subregions) governs the possibility of having locally various functional dynamics.

However, there is an additional concept often recalled in the literature to describe the diffusion of energy which is the mean free path. We believe, on the basis of the physical description presented here, (i.e., that a protein can be seen as a set of potentials wells) that this particular vision of transport is erroneous and here are the reasons for this. The mean free path considers that energy is exchanged through quasiparticles propagating for a certain time or along a certain distance before undergoing a collision inducing a loss of phase of the wave packet and leading in some cases to energy loss or relaxation mechanisms. This view implies that the size of the system is very large compared to the coherence length of the wave packets describing these quasiparticles. We have shown here that the localization is an interferential phenomenon, where the waves propagating in the complex medium will create reflection/transmission phenomena giving rise to the localization spots. Clearly in this type of regime, the coherence length of the waves is of the order of the size of the system otherwise no localization phenomenon could be observed. We therefore believe that the transport in the protein is not a classical scattering phenomenon but rather a hopping transport phenomenon, induced by the set of weak or resonant couplings between localizing subdomains resulting from wave interferences.

### Summary of the Main Results

Bilocalization, as it is presented here, is a theory that allows to describe in a condensed and quite robust way the dynamics of proteins, that is the evolution of the positions of the degrees of freedom in space and in time, at the two limits of the slowest and fastest time scales. This tool can be adapted to any cohesion model, atomistic or coarse-grained. If it is well known that a protein is a dynamical object with multiscale behaviors, there are in fact no tools that surpass the traditional normal mode analysis to highlight the domains characterizing the motions at a given frequency/time period. The normal mode approach widely developped in the litterature, is neither rigorous nor sufficient to establish a complete picture of proteins and enzymes dynamics. Indeed, computing eigenmodes consists in accessing to the vector space that allows to decompose each motion with a well defined orthonormal basis. In other words, the motion of each atom/AA is a linear combination of the eigenmodes. If it is possible to access to this basis by solving an eigenvalue problem, we learn nothing about the combination of these modes (i.e., we do not know the coefficients that define the linear combination, nor the phase shifts between these modes), thus the information we have access to is partial, and one cannot rigorously predict an observed motion on the sole knowledge of the eigenvectors. In addition, if we want to study large systems like the SPIKE protein or RNA-polymerase (roughly 5000 AA), it is almost unmanageable to manipulate 15 0,000 eigenvalues with eigenvectors distributed over 50,000 amino acids. In most works, the analysis is often restricted to the few lowest energy mode, which form a too small fraction of the full eigenmodes spectrum. By solving the linear system *LU* = 1, with the right localization operator *L*, *i.e*,*.* by extracting a single quantity, associated to each degree of freedom, one gets a more accurate information roughly 100 times faster to calculate because solving a linear system is much easier in terms of algoritmic complexity than calculating the full spectrum of an operator.

The analysis proposed here is not only interesting to predict rigorously the predominant motions observed in these systems, but it also allows to understand the way energy flows in the latter. That is, which domains are energetically coupled and which are isolated (Chalopin et al., 2019).

In addition, bilocalization also offers a deep description of the underlying physical mechanisms that allow to universalize the formation of a partitioning/confinement of vibrations in proteins’ domains. To this end, we have established an analogy with quantum mechanics by showing that the structural disorder of the protein, which translates into an inhomogeneity of its rigidity, produces a confinement potential for the phonons (vibration modes) of the system just as a crystal produces a potential barrier for an electron (Filoche et al.). We will see later on, how this physical description allows to unify the set of well known methods such as the analysis of proteins by contact network, the long range transmission mechanisms induced by rigidity, which are in fact metrics that have some efficiency, but do not share any common physical picture of the exchange processes involved.

## Conclusion

We have developed a theory to link the topological structure of a molecule (genetically encoded) and its function by studying the (coherent) motions imposed by its complex form. We have shown that structural disorder produces *de facto* a partitioning of its slow and fast motions into several molecular subdomains. Two complementary effective confinement potentials predict these features. At the energy equipartition, each molecular domain is identified by a localized motion of characteristic frequency. This one to one correspondence between topological irregularities and dynamical patterns allows the exploration of large dynamical variability. Regarding the dynamics of a homogeneous collection of harmonic oscillators, disorder/randomness thus appears as a prerequisite to produce a function, that is to say, an efficient temporal and spatial coordination of atomic motions, suited to accomplish a function in a particular environment. This quantitative analysis of this structure-function dynamical interplay forms the basis of a new paradigm for understanding how nature accomplishes molecular design, that is to say, from which generator of properties, evolution manages to select biological functions from an easily modifiable reservoir of dynamical patterns. These localizing properties can be observed experimentally by looking at the rigidity (or its temperature factor) or by measuring the thermal transport which takes place in these systems and which must appear as highly anisotropic, due to the percolation phenomena produced by the effective confinement potential produced by the disorder.

## Data Availability

The original contributions presented in the study are included in the article/Supplementary Material, further inquiries can be directed to the corresponding author.
